# Study on the One-Dimensional Impact Compression Characteristics of Coral Sand Under Conditions of Different Relative Density

**DOI:** 10.3390/ma18051113

**Published:** 2025-02-28

**Authors:** Xin Liu, Huiqi Ren, Hui Zhang, Zhi Yi

**Affiliations:** 1 Defense Engineering Institute, Academy of Military Sciences, Luoyang 471023, China; lx252606@163.com (X.L.); freeyogism@163.com (Z.Y.); 2 Xi’an High-Tech Research Institute, Xi’an 710025, China; 3 School of Intelligent Construction and Civil Engineering, Luoyang Institute of Science and Technology, Luoyang 471023, China; huizhang0821@163.com

**Keywords:** stress–strain relationship, relative density, bearing capacity

## Abstract

Dynamic compression tests under quasi-one-dimensional strain conditions were conducted on coral sand with different relative densities using the Split Hopkinson Pressure Bar (SHPB) apparatus. The experimental results indicate that the stress–strain curves of coral sand exhibit approximately three basic forms, each encompassing three distinct stages: skeletal sliding and yielding, compaction, and unloading. The occurrence of the lock-up phenomenon is jointly influenced by relative density, strain rate, and moisture content. A higher moisture content, lower relative density, and larger strain rate tend to facilitate the occurrence of the lock-up phenomenon. The influence of relative density on the compression behavior of coral sand is comprehensively affected by both the moisture content and the strain rate. Under dry or low-saturation conditions, as the relative density increases, both the tangent modulus in the first stage of the stress–strain curve and the pre-consolidation pressure exhibit enhancements of varying degrees, and the specimens’ bearing capacity continues to improve. Conversely, under conditions of high saturation and low strain rate, an increase in relative density results in a decrease in the specimens’ bearing capacity. However, as the strain rate progressively increases, the specimens’ bearing capacity will improve continuously, swiftly transitioning from a state of decrease to increase, with the magnitude of this improvement continually intensifying.

## 1. Introduction

Coral sand, also known as calcareous soil or calcareous sand, is a special type of biodetrital sediment characterized by porosity, heterogeneity, discontinuity, and particle fragility. Its mechanical properties differ significantly from those of traditional terrestrial quartz sand, making it a key scientific issue in coral reef engineering research [1,2,3]. Current research on calcareous sand primarily focuses on its basic physical and mechanical properties and static mechanical characteristics [1,2,3,4,5], particle crushing [1,6,7,8,9,10,11,12,13,14,15], and dynamic properties under complex stress conditions or cyclic loading [16,17,18,19,20]. However, from the perspectives of disaster prevention and control, the study of the dynamic mechanical properties of coral sand under high strain rates is crucial.

According to Omidvar et al. [21], numerous factors influence the dynamic mechanical properties of sandy soil, including initial porosity/density, saturation (moisture content), and particle characteristics (such as particle shape, size, gradation, surface structure, and mineralogy). Dong et al. [22] experimentally observed that coral sand exhibits a significant strain rate effect within the strain rate range of 460 to 1300 s^−1^, with strain rate sensitivity primarily related to internal pores of coral sand particles and inter-particle friction. Wei et al. [23,24] conducted comparative studies on the dynamic mechanical properties of calcareous sand and Fujian standard sand under impact loading, finding that both sands exhibited minimal strain rate effects within the studied strain rate range. The compressibility of unsaturated quartz sand increased with moisture content, whereas unsaturated calcareous sand was insensitive to moisture content, and when the soil reached saturation, the main bearing medium became pore water, making the specimen less compressible. Wen et al. [25] experimentally investigated the dynamic mechanical properties of confined calcareous sand under quasi-static and dynamic loading, finding that pre-compression has a limited influence on the mechanical properties of calcareous sand. Strain rate effects exist during volumetric compression, and static and dynamic volumetric deformation relationships for calcareous sand were provided. Lv et al. [26,27,28,29,30,31,32,33] conducted a systematic study on the Split Hopkinson Pressure Bar (SHPB) experimental technique for calcareous sand and its dynamic mechanical properties. They presented dynamic characteristic parameters such as compressive modulus, yield stress, and compression index for one-dimensional compression of calcareous sand under high strain rates. Their findings indicated that under impact loading, the stress–strain relationship of calcareous sand approximates linearity, with no evident hardening phenomena arising from particle deformation, yield of the granular skeleton, and particle sliding. The axial stress–strain response exhibits minimal sensitivity to strain rates. The compressive modulus increases with the enhancement of relative density. Particle crushing occurs throughout the loading process, and the influence of relative density on particle crushing is limited. Zhao et al. [34] experimentally found that the tangential modulus of wet samples is higher than that of dry samples when the strain of calcareous sand is less than 0.025, but the opposite is true when the strain exceeds 0.025. The tangential modulus of wet calcareous sand first decreases and then increases with increasing moisture content. They also proposed a model for the lock-up phenomenon in unsaturated calcareous sand.

Currently, research on the dynamic mechanical properties of coral sand under high strain rates has yielded certain achievements. However, a comprehensive understanding remains elusive. For instance, there is no unified consensus on whether coral sand exhibits a strain rate effect or a specific influence pattern of moisture content on the compressibility of calcareous sand. Extensive studies have been conducted on the mesoscopic crushing behavior of coral sand particles with varying particle characteristics. However, research on the dynamic macroscopic response of coral sand under the coupled influence of factors such as initial porosity/density, saturation (moisture content), and other parameters requires further in-depth exploration. Existing constitutive models for coral sand are far from perfect, and multiscale models that comprehensively consider multiple factors including initial porosity/density, saturation (moisture content), particle characteristics, and their interactions are also scarce. Therefore, enhancing the understanding of the dynamic response characteristics of coral sand under impact loading and revealing the coupled interaction patterns and mechanisms through which factors such as initial porosity/density and saturation (moisture content) affect these characteristics are of great significance.

In this study, the SHPB experimental apparatus was utilized to conduct quasi-one-dimensional strain dynamic compression tests on coral sand with different relative densities. A systematic analysis was conducted on the macroscopic dynamic response laws of coral sand, including the yield stress and compression index, as well as the influence patterns and underlying mechanisms of relative density on the one-dimensional impact compression characteristics of coral sand.

## 2. Experimental Setup and Specimens

### 2.1. Experimental Setup

**Figure 1:**
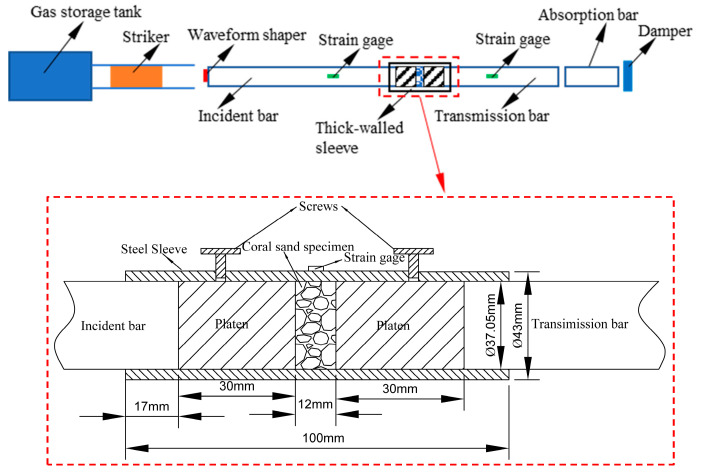
SHPB experimental setup and thick-walled sleeve (confining ring).

The SHPB experimental setup (Defense Engineering Institute, Academy of Military Sciences, Luoyang, China) used in this study, as illustrated in Figure 1, is mainly composed of a gas storage tank, striker, incident bar, transmission bar, absorption bar, and damper. As in many similar studies [22,23,24,25,35,36,37,38], the stress and strain history curves of the specimens were measured using strain gauges attached to the incident and transmission bars. The striker, incident bar, and transmission bar are made of an aluminum alloy with a diameter of 37 mm and lengths of 800 mm, 4000 mm, and 3000 mm, respectively. The material properties of the bars and striker include a density of 2.7 g/cm^3^, an elastic modulus *E*_0_ of 70 GPa, and an elastic longitudinal wave velocity *C*_0_ of approximately 5092 m/s. A thick-walled sleeve made of high-strength 30CrMnSiA alloy steel (with an elastic modulus of 196 GPa and a Poisson’s ratio of 0.3) is fixed radially around the specimen, with an inner diameter of 37.05 mm and an outer diameter of 43 mm. The thick-walled sleeve aims to provide confining pressure to the specimen and control its initial relative density. To ensure dynamic stress equilibrium and a uniform strain rate in the specimen, and following practices in numerous similar studies [22,23,24,25,35,36,37,38], a waveform shaper made of rubber is attached to the end of the incident bar in this study. This extends the rise time of the loading pulse and eliminates wave oscillations caused by dispersion effects. Additionally, the specimen length is appropriately controlled at 12 mm. The striker impacts the incident bar at a certain velocity to generate a stress pulse, and the stress–strain curve of the specimen is obtained by measuring the strain pulses in the incident and transmission bars and performing subsequent calculations.

In the experiments, the pressure in the gas storage tank of the SHPB system was set to 0.2 MPa, 0.4 MPa, 0.6 MPa, and 0.8 MPa, respectively, corresponding to striker velocities of approximately 3.4 m/s, 5.22 m/s, 6.75 m/s, and 8.1 m/s. The aim was to provide four impact intensities, resulting in four different strain rates of varying magnitudes in the coral sand specimens.

### 2.2. Specimen Preparation

**Figure 2:**
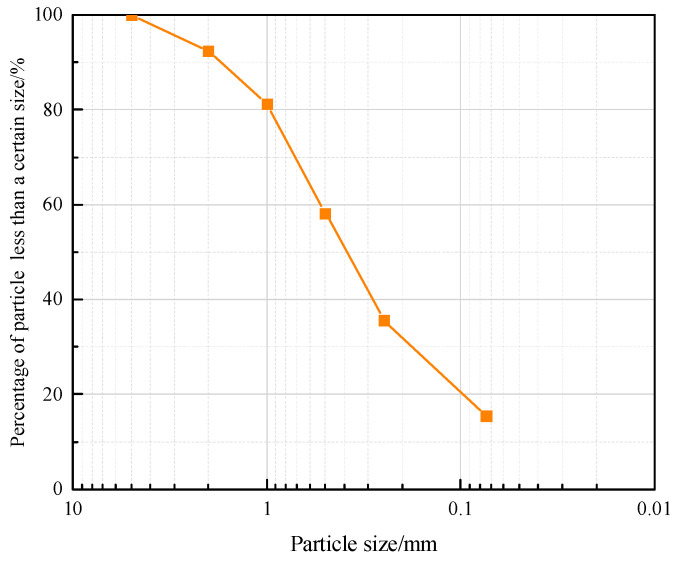
Grading curve of calcareous sand [39].

The coral sand samples used in this study are from the same batch as those used in the previously reported literature [39], whose gradation curve is shown in Figure 2. The average particle size of the calcareous sand is 0.43 mm, the non-uniformity coefficient is 10.10, and the curvature coefficient is 1.29. The maximum and minimum dry densities of the coral sand specimens are 1.536 g/cm^3^ and 1.183 g/cm^3^, respectively, with a specific gravity of 2.78. To investigate the influence of relative density on the dynamic response characteristics of coral sand, three relative densities of 30%, 60%, and 90% were established in this study. According to the calculation formula for relative density (Equation (1)), the corresponding dry densities of the specimens for these three relative densities are approximately 1.27 g/cm^3^, 1.37 g/cm^3^, and 1.49 g/cm^3^, respectively. Subsequently, the mass of the coral sand specimens for each relative density can be determined based on the volume of the specimen container.

(1) $$D_{r}=\frac{\rho_{d\max}(\rho_{d}-\rho_{d\min})}{\rho_{d}\left(\rho_{d\max}-\rho_{d\min}\right)}$$

wherein $D_{r}$ denotes the relative density, $\rho_{d\max}$ denotes the maximum dry density, $\rho_{d\min}$ denotes the minimum dry density, and $\rho_{d}$ denotes the dry density of the specimen.

Various moisture contents of 0.5, 0.3, 0.2, and 0.1 were set in the experiments. To prepare the moist specimens, a specific amount of water was added to the dry specimens according to Equation (2) and thoroughly mixed until uniform.

(2) $$w=(\frac{m_{0}}{m_{d}}-1)\times 100\%$$

In Equation (2), w denotes the moisture content, $m_{0}$ denotes the total mass of the sandy soil, and $m_{d}$ denotes the mass of the dry sand.

The test conditions for the SHPB experiments are shown in Table 1, with each condition being repeated at least once effectively. In Table 1, the first digit in the test number represents the relative density $D_{r}$, the second digit represents the moisture content w, the third digit represents the pressure in the gas storage tank in the SHPB system, and the last number ‘*i*’ represents the *i*th test conducted under the specific operating condition. For example, the test number 0.6-0.3-0.8-1 represents the first experiment conducted under operating conditions with a relative density of the coral sand sample of 0.6, a moisture content of 0.3, and a gas pressure for the gas storage tank of 0.8 MPa.

Coral sand is a typical granular medium, and issues related to specimen molding must be considered before conducting experiments. Moreover, its strength is correlated with hydrostatic pressure, necessitating a certain degree of lateral confinement for SHPB testing. As in numerous references [22,23,24,25,26,27,34,36,37,40], this study also employs a thick-walled sleeve (confining ring) combined with upper and lower aluminum alloy blocks to impose lateral rigid confinement on the coral sand specimens. As shown in Figure 1, the aluminum alloy blocks have flat ends, an outer diameter of ∅37 mm, and a height of 30 mm. They can slide axially along the inner wall of the thick-walled cylinder and are made of the same material as the incident and transmitted bars of the 37 mm SHPB device. The spacer blocks have a height of 11 mm and an inner diameter of 37 mm, primarily serving to reserve a certain height below the steel blocks to facilitate the positioning of the transmitted bar during the experiment. Under the rigid confinement of the thick-walled cylinder, the stress state of the specimen during deformation can be regarded as a quasi-one-dimensional strain state, which is similar to the stress state of the medium in the near field during penetration or explosion problems, thereby possessing significant research significance.

**Figure 3:**
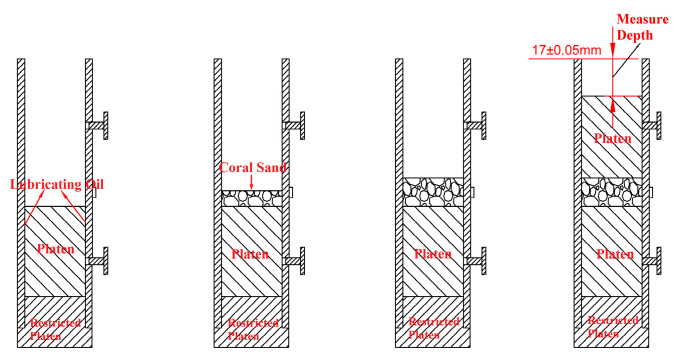
Schematic diagram of the specimen preparation process.

The specimen preparation process is illustrated in Figure 3. First, the spacer block, lower aluminum alloy block, and thick-walled cylinder are placed sequentially on a platform. The aluminum alloy block and thick-walled cylinder are in close contact and lubricated with Vaseline. Then, a quantitative amount of coral sand is weighed. A layered sampling method is adopted to load the sample. The specimen is divided into three equal parts and loaded into the sleeve in three layers. After loading the first and second layers of the specimen, the surface of the coral sand is leveled, and the thick-walled cylinder and sand sample are lightly tapped with a rubber mallet. After loading the third layer of the specimen, the surface is leveled, and the upper aluminum alloy block is placed. The thick-walled cylinder and upper aluminum alloy block are lightly tapped with a rubber mallet to ensure that the number and force of taps on the thick-walled cylinder and specimen or aluminum alloy block are basically consistent after each layer of coral sand is loaded into the sleeve, thereby ensuring that the specimen is basically uniform along its length. When compacting the third layer of the specimen, a vernier caliper is used to measure the height h from the surface of the upper aluminum alloy block to the upper-end face of the sleeve while tapping. The height of the coral sand specimen is controlled through multi-point measurements, thereby controlling the relative density of the coral sand specimen. The surfaces of the upper and lower aluminum alloy blocks are adjusted to be parallel. When h is 17 ± 0.05 mm, specimen preparation is deemed satisfactory, and then the upper and lower aluminum alloy blocks are tightened with preset bolts. During the experiment, the sleeve is placed between the incident bar and the transmitted bar, ensuring that the upper and lower aluminum alloy blocks are aligned with the incident bar and transmitted bar, respectively, and maintain good contact. Before the experiment begins, the preset bolts are loosened to allow the upper and lower blocks to slide freely.

## 3. Results and Discussion

### 3.1. Validity of Experimental Results

During the SHPB test, the stress equilibrium at the front- and rear-end faces of the specimen serves as a crucial criterion for assessing the validity of the test data. This paper takes the test numbered 0.3-0.5-0.8-1 as an example to demonstrate the validity of the experimental results presented herein.

Based on the principles of the SHPB test, the stress $\sigma_{F}\left(t\right)$ experienced by the front-end face of the coral sand specimen is given by:

(3) $$\sigma_{F}\left(t\right)=\left[\sigma_{i}\left(t\right)+\sigma_{r}\left(t\right)\right]\alpha_{1}$$

where $\sigma_{i}\left(t\right)$ and $\sigma_{r}\left(t\right)$ are the incident wave and reflected wave on the incident bar, respectively, and $\alpha_{1}$ is the ratio of the cross-sectional area of the incident bar to that of the specimen. The stress $\sigma_{R}\left(t\right)$ experienced by the rear-end face of the coral sand specimen is given by:

(4) $$\sigma_{R}\left(t\right)=\sigma_{t}\left(t\right)\alpha_{2}$$

where $\sigma_{t}\left(t\right)$ is the transmitted wave on the transmitted bar and $\alpha_{2}$ is the ratio of the cross-sectional area of the transmitted bar to that of the specimen. In this study, both $\alpha_{1}$ and $\alpha_{2}$ are approximated to 1. Therefore, the validity of the test results can be assessed by comparing the consistency between the incident wave plus the reflected wave on the incident bar and the transmitted wave on the transmitted bar.

**Figure 4:**
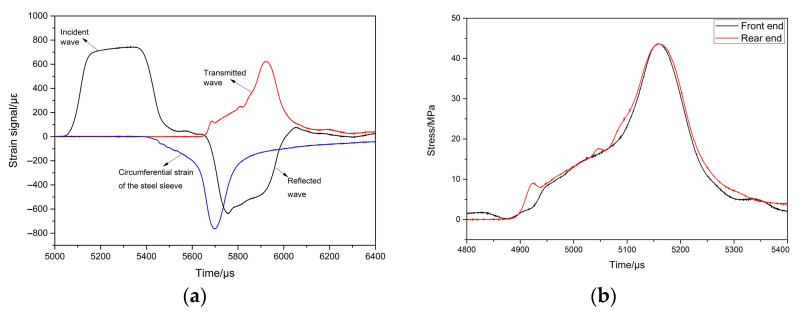
Typical records of SHPB test results of test number 0.3-0.5-0.8-1 and its dynamic equilibrium check under confinement by steel tubing. (**a**) Measured strain signal; (**b**) Stress comparison between the front and rear ends of the sample.

Figure 4a displays the measured strain signals from the strain gauges on the incident bar, transmitted bar, and thick-walled cylinder, and the corresponding stress comparisons at the front- and rear-end faces of the specimens for the test numbered 0.3-0.5-0.8-1 are illustrated in Figure 4b. From Figure 4, it can be observed that after adding a pulse shaper, the rising edge time of the incident shock wave is approximately 132 μs, with a plateau stage during the peak phase. There is a certain time interval between the incident wave and the reflected wave. Except for the initial loading stage, the stresses at the front- and rear-end faces of the specimen are basically consistent, indicating that the dynamic equilibrium state of the specimen is good and the test results are valid.

### 3.2. Typical Test Results

**Figure 5:**
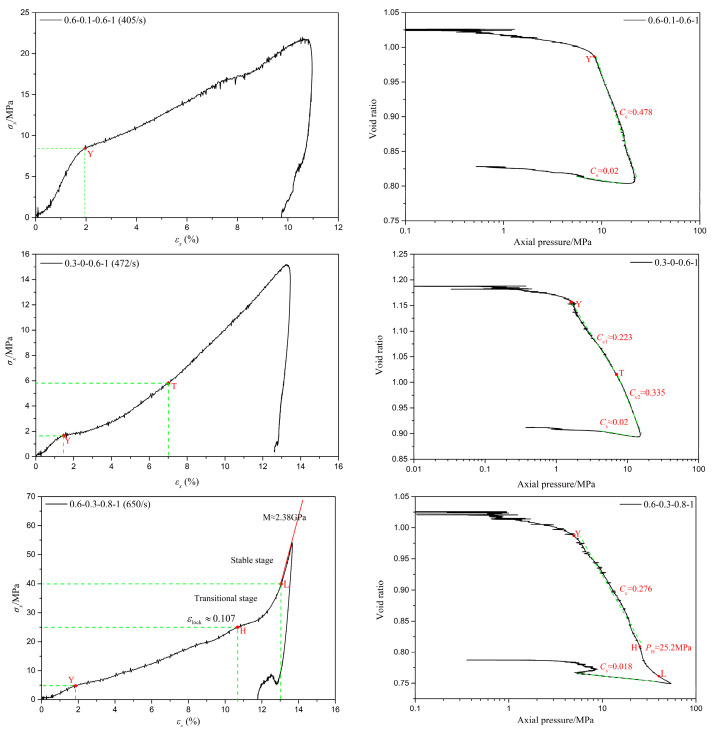
Stress–strain and compression curves of typical coral sand.

Analysis of the test results revealed that the stress–strain curves of coral sand generally exhibited three basic forms. A typical test result from each basic form was selected for analysis, as shown in Figure 5. These include the stress–strain curves and corresponding compression curves (e−logp curves in a semi-logarithmic coordinate system) of coral sand samples obtained from tests 0.6-0.1-0.6-1, 0.3-0-0.6-1, and 0.6-0.3-0.8-1, respectively. The void ratio e is:

(5) $$e=e_{0}-\left(1+e_{0}\right)\varepsilon_{v}$$

where $\varepsilon_{v}$ denotes the volumetric strain, which approximately equals the axial strain $\varepsilon_{x}$ in a one-dimensional strain state.

(6) $$e_{0}=\frac{G_{s}\left(1+w\right)\rho_{w}}{\rho }-1=\frac{G_{s}\rho_{w}}{\rho_{d}}-1$$

where $G_{s}$ denotes the specific gravity of coral sand, w denotes the moisture content, $\rho_{w}$ denotes the density of water, typically taken as 1 g/cm^3^, ρ denotes the density of the coral sand sample, and $\rho =\left(1+w\right)\rho_{d}$, and $\rho_{d}$ denote the initial dry density of coral sand. p denotes axial pressure, and $p=\left|\sigma_{x}\right|$, $\sigma_{x}$ denotes axial stress [36,37].

$\varepsilon_{x}$ and $\sigma_{x}$ can be calculated according to Equation (7).

(7) $$\left\{ \begin{array}{l} \sigma_{x}\left(t\right)\approx 0.5E_{0}\left[\varepsilon_{i}\left(t\right)+\varepsilon_{r}\left(t\right)+\varepsilon_{t}\left(t\right)\right]\\ \varepsilon_{x}\left(t\right)\approx \left(C_{0}/L_{s}\right)\int_{0}^{t}\left[\varepsilon_{i}\left(t\right)-\varepsilon_{r}\left(t\right)-\varepsilon_{t}\left(t\right)\right]dt \end{array}\right.$$

where $L_{s}$ is the initial height of the calcareous sand sample, and its value is 12 mm, $\varepsilon_{i}\left(t\right)$, $\varepsilon_{r}\left(t\right)$ are, respectively, the incident strain and reflected strain measured by the strain gauge on the incident bar, and $\varepsilon_{t}\left(t\right)$ is the transmitted strain measured by the strain gauge on the transmission bar.

As illustrated in Figure 5, based on the test results of 0.6-0.1-0.6-1, the stress–strain curve of coral sand in its first basic form exhibits three distinct stages. The first stage, characterized by skeletal slippage and yield, begins at the origin and terminates at an inflection point ‘Y’. During this stage, the stress–strain relationship and the corresponding compression curve approximate a linear relationship. The coral sand specimen undergoes primary skeletal slippage, rotation, and buckling deformation, with gases and water being expelled from the pores and particles rearranging themselves. The second stage, known as the compaction stage, starts from the inflection point ‘Y’ and concludes at the peak stress point. The stress–strain relationship and the corresponding compression curve in this stage also approximate a linear relationship, albeit with a different slope compared to the first stage. The inflection point ‘Y’ on both lines corresponds to the maximum curvature on the compression curve, marking the yield point of granular materials [36,37]. Consequently, during the second stage, the coral sand specimen primarily undergoes deformation from particle crushing. After crushing, the fragmented particles fill the voids between the original particles and temporarily remain unstressed. However, as shear deformation progresses, the particles rearrange their positions, further compacting the specimen. The temporarily unstressed small particles may then undergo additional stress and continue to fragment into finer particles. Therefore, the stress–strain curve exhibits slight oscillations as the specimen undergoes gradual compaction. The third stage is the unloading stage, which begins at the peak stress point and continues until the end. During this stage, the stress–strain relationship approximates a linear decrease, with stress rapidly unloading and strain slightly decreasing as stress diminishes, indicating a degree of expansion in the specimen. The corresponding compression curve is approximately linear during the initial stages of unloading. Consistent with the literature [37], pre-consolidation pressure *P*c is defined as the stress required to induce the compaction of sandy soil at the transition between the two linear segments of the compression curve on a semi-logarithmic plot. The compression index *C*c represents the slope of the linear segment of the e−logp curve after pre-consolidation, while the swelling index *C*_S_ is the slope of the linear segment during the initial unloading stage of the e−logp curve, describing the expansion of the coral sand specimen during unloading. For test 0.6-0.1-0.6-1, *P*_c_ is approximately 8.4 mPa, *C*_c_ is approximately 0.478, and *C*_S_ is approximately 0.02.

The stress–strain curve of the second basic form of coral sand, as demonstrated by the test results of 0.3-0-0.6-1, exhibits similarities to that of the first basic form. However, a notable difference lies in the second stage, or the compaction phase, where the stress–strain relationship approximates an exponential relation. As strain increases, the compression curve deviates from a simple straight line, initially deflecting gradually towards the vertical axis of the coordinate system, with the slope of the curve gradually increasing. Due to the modest rate and magnitude of this increase, the curve can be approximately simplified as a straight line. Upon reaching a turning point ‘T’, the slope of the curve remains almost constant, and the compression curve resumes a linear form. This indicates that starting from an inflection point ‘Y’, particle crushing within the specimen initiates, accompanied simultaneously by skeletal sliding, rotation, and buckling. As the specimen undergoes gradual compaction, skeletal sliding, rotation, and buckling, deformations decrease, while particle crushing becomes more prevalent. Beyond point ‘T ’ (approximately 5.8 mPa), deformation from particle crushing dominates the deformation behavior of the specimen. Similar to the aforementioned context, the stress corresponding to inflection point ‘Y’ at the transition between the first and second stages of the compression curve on a semi-logarithmic plot is defined as the pre-consolidation pressure *P*_c_. The slope of the approximate linear segment and the linear segment of the curve beyond pre-consolidation point ‘Y’ is designated as the compression index *C*_c_. For test 0.3-0-0.6-1, *P*_c_ is approximately 1.65 MPa, *C*_c1_ is approximately 0.223, *C*_c2_ is approximately 0.335, and *C*_S_ is approximately 0.02.

The stress–strain curve of the third basic form of coral sand, as illustrated by the results of test 0.6-0.3-0.8-1, also resembles the stress–strain curve of the first basic form. However, a third stage is introduced between the compaction and unloading phases. This stage begins at point ‘H’, the starting point where the stress–strain curve begins to rise sharply, and ends at the peak stress point. During this stage, the stress–strain relationship first follows an exponential pattern, with stress increasing rapidly with strain, indicating that the specimen is gradually hardening. When the strain increases to approximately 0.13, the rate of stress increase stabilizes to a constant value, and the stress–strain relationship becomes linear. Similar to the relevant literature, the phenomenon of a sudden increase in stiffness in coral sand due to “full saturation” is termed the lock-up phenomenon. The strain value at which the tangent modulus of the stress–strain curve increases sharply and the constant value of this tangent modulus are defined as the lock-up strain $\varepsilon_{lock}$ and lock-up modulus M, respectively [34,41]. For test 0.6-0.3-0.8-1, the lock-up strain $\varepsilon_{lock}$ is 0.107 and the lock-up modulus M is approximately 2.38 GPa, which is basically consistent with the research result of 2.34 GPa by Zhao et al. [41]. For coral sand with a relative density of 0.6 and a moisture content of 0.3, the calculated theoretical lock-up strain, representing the ratio of air volume in the sample, is 0.095, which is relatively close to the experimental value of 0.107. According to the research findings in the relevant literature, the stiffness of water at around 50 MPa is approximately 3.16 GPa [42], which is approximately 25% higher than the lock-up modulus of 2.38 GPa. The primary reason for this phenomenon is that coral sand, characterized by high porosity, still experiences the compression of pore air and the expulsion of pore water during the compression process after lock-up strain. This also indicates that the compression of coral sand after lock-up strain is not solely controlled by the compression of pore water. Based on the characteristics of the axial stress–strain curve, the third stage, or lock-up phenomenon stage, can be further divided into a transition stage with a rapidly increasing tangent modulus and a stable stage with a constant tangent modulus, which is also consistent with the results of the related literature [34,41]. For test 0.6-0.3-0.8-1, the transition phase spans from point ‘H’ to point ‘L’, while the stable phase extends from point ‘L’ to the peak stress point. Meanwhile, for test 0.6-0.3-0.8-1, the pre-consolidation pressure *P*c is approximately 4.95 MPa, the compression index *C*c is approximately 0.276, and the swelling index *C*_S_ is approximately 0.018.

**Figure 6:**
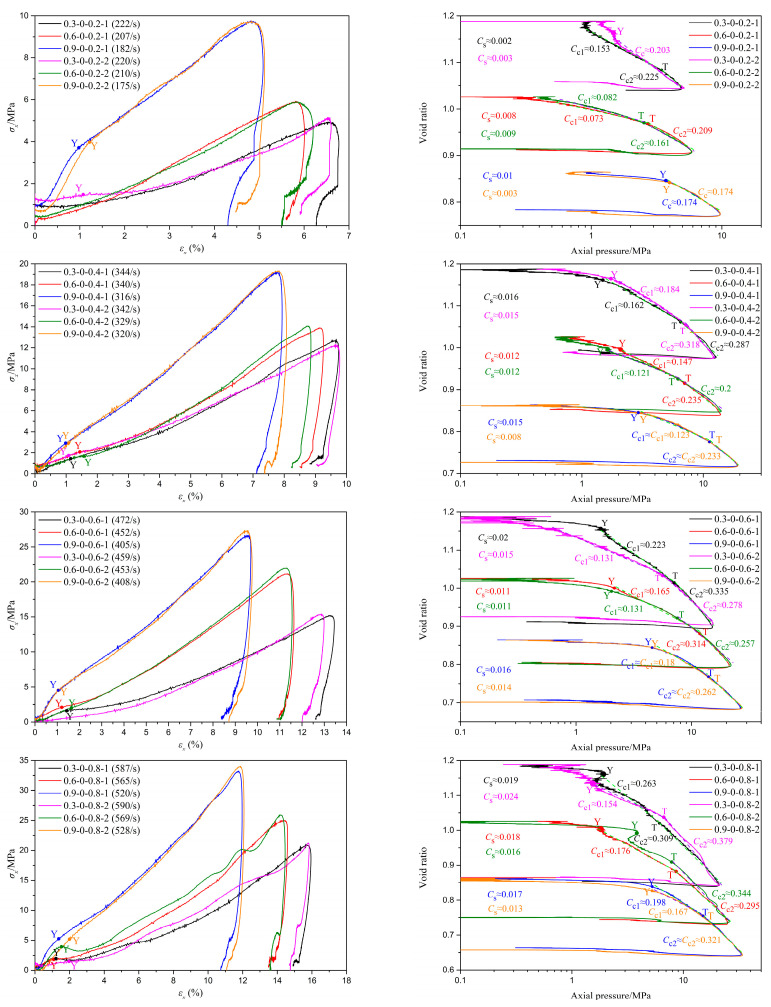
Influence of relative density on the impact compression performance of coral sand under dry conditions.

**Figure 7:**
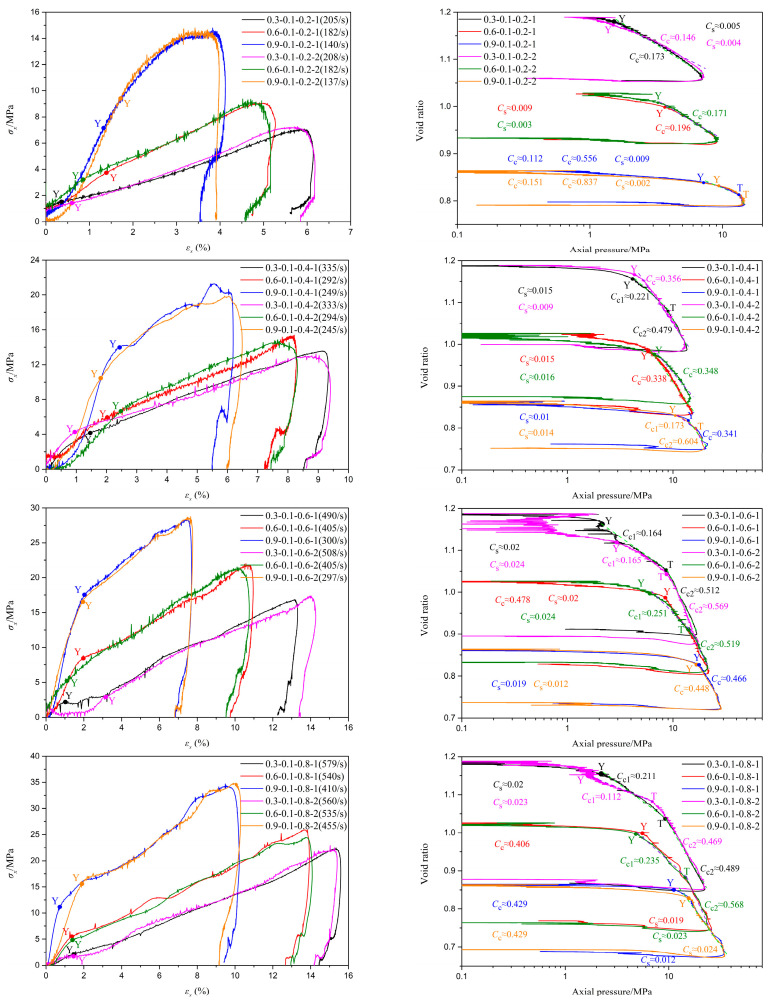
Influence of relative density on the impact compression performance of coral sand at a moisture content of 0.1.

**Figure 8:**
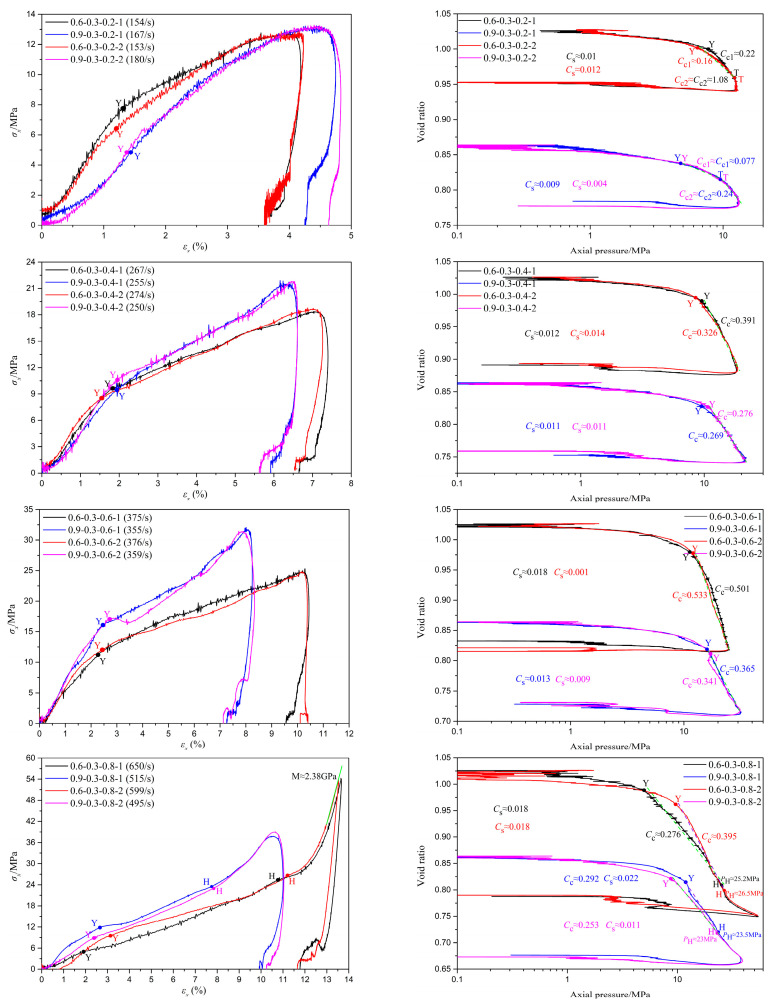
Influence of relative density on the impact compression performance of coral sand at a moisture content of 0.3.

Figure 6, Figure 7 and Figure 8 depict the stress–strain curves and their corresponding compression curves for coral sand samples with different relative densities, under dry conditions, 10% moisture content, and 30% moisture content, respectively, and the corresponding parameters of compressibility are listed in Table 2. It should be noted that in some experiments, the stress initiates from a non-zero value. This is attributed to the slight superposition of the reflected wave head and the incident wave tail. However, throughout the entire loading process, the stress at the front and rear ends of the sample remains balanced. Therefore, it can be considered that the test results with a non-zero starting stress are equally effective.

**Figure 9:**
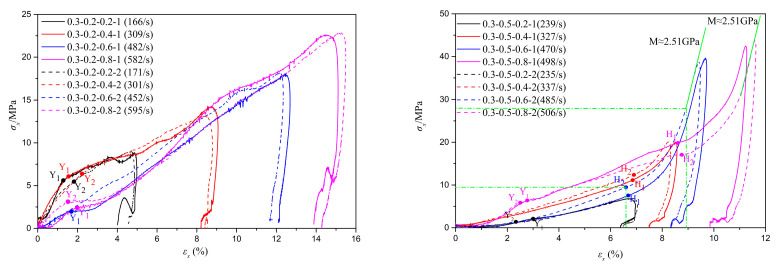
Stress–strain curves of coral sand at a relative density of 0.3 and moisture content of 0.2 and 0.5.

It is evident from the figures that the second basic form of the stress–strain relationship mentioned earlier primarily occurs under conditions of low relative density and no moisture content, such as tests 0.3-0 and 0.6-0. Among them, the results of tests 0.3-0-0.2-1 and 0.6-0-0.2 exhibit slight deviations from the typical test 0.3-0-0.6-1, as there is no pronounced inflection point ‘Y’ in the stress–strain curves. This indicates that particle crushing of coral sand does not occur instantaneously in large quantities under low strain rates. The primary reason for this phenomenon is that, due to the low deformation strain rate and high porosity of the specimens, skeletal compaction deformation caused by particle sliding and rotation can occur over a larger strain range, continuously consuming a significant amount of energy and thereby preventing instantaneous massive particle crushing. Combined with Figure 9, it can be observed that the third basic form of the stress–strain relationship predominantly appears under conditions of high strain rates and high moisture contents, such as tests 0.3-0.5-0.6, 0.3-0.5-0.8, 0.6-0.3-0.8, and 0.9-0.3-0.8. The probable cause of this phenomenon is that as the strain rate and moisture content gradually increase, the degree of compression deformation of coral sand also gradually increases, potentially leading to the lock-up phenomenon upon reaching a certain threshold, thereby exhibiting the third basic form of the stress–strain relationship. Stress–strain curves under other conditions are mainly characterized by the first basic form. It should be noted that under certain conditions of low relative density with low moisture content, or high relative density without moisture, such as in tests 0.9-0-0.4 and 0.3-0.1-0.4, the compression curves deviate slightly from the typical test, 0.6-0.1-0.6-1. Specifically, in the compression segment, the compression curves do not exhibit a simple linear relationship but are similar to the second fundamental form of stress–strain curves. The primary reason for this phenomenon is that under conditions of low relative density with a small amount of water or high relative density without water, the degree of compression at the yield point of the samples is limited, leaving a certain amount of porosity. Therefore, after passing the yield point, particle breakage occurs concurrently with skeleton slip, rotation, and buckling deformation. As the samples gradually become more compact, skeleton slip, rotation, and buckling deformation gradually decrease, while particle breakage gradually increases. Beyond point ‘T’, the deformation of the samples is predominantly controlled by particle breakage. The above conclusions demonstrate that the stress–strain relationship of coral sand is comprehensively influenced by various factors, including relative density, moisture content, and strain rate.

### 3.3. The Influence of Relative Density

The primary contact between coral sand particles is point contact. Due to variations in compactness, the number and contact area of point contacts between particles in coral sand samples differ, leading to significant differences in the stress acting at individual contact points. Consequently, coral sand samples with different relative densities exhibit distinct mechanical behaviors under identical conditions, resulting in varying impact compression performances.

As illustrated in Figure 6, Figure 7, Figure 8 and Figure 9 and Table 2, under conditions of zero moisture content and 0.1 moisture content, it is observed that as relative density increases, both the tangent modulus and pre-consolidation pressure in the first stage of the stress–strain curve exhibit varying degrees of increase, except for a few isolated tests influenced by experimental errors or inherent random differences among samples. Notably, the peak stress experienced by the coral sand specimens undergoes a significant augmentation, and the stress corresponding to the same strain exhibits an overall increase of varying magnitudes. Concurrently, there is a marked reduction in both peak strain and ultimate deformation. Taking the experiment with a moisture content of 0.1 and a gas pressure in the storage tank of 0.8 MPa as an example, as the relative density increases, the tangent moduli in the first stage of the stress–strain curves are approximately 131 MPa, 369 MPa, and 1257 MPa, respectively. The pre-consolidation pressures are approximately 1.9 MPa, 5.14 MPa, and 13.27 MPa, respectively, while the peak stresses are approximately 22 MPa, 25.3 MPa, and 34.5 MPa, respectively. The corresponding peak strains are approximately 15.6%, 14%, and 10.2%, respectively. These results indicate a continuous improvement in the bearing capacity of the tested samples. The primary reasons for these changes are attributed to the fact that, under identical conditions, coral sand specimens with a higher relative density have a smaller porosity ratio, a larger coordination number of particles, and a larger contact area between particles, resulting in less contact stress under the same conditions and greater difficulty in reaching the critical stress for particle breakage. Therefore, the higher the relative density, the greater the pre-consolidation pressure and the stress experienced at the same strain. Under low pressure, as stress increases, original contacts gradually fail, and coral sand particles undergo sliding, rotation, or bending deformation, displacing to a denser and more stable equilibrium position. The magnitude of displacement or compression depends on the inter-particle friction resistance against displacement between coral sand grains. The higher the coral sand density, the greater the resistance to displacement of coral sand grains, leading to less compressive deformation. Under high pressure, due to the higher density of coral sand, the contact stress between particles decreases, making breakage more difficult and thus reducing the amount of breakage. Therefore, the greater the relative density, the higher the peak stress of the sample and the smaller the peak strain and final deformation.

Under the condition of a moisture content of 0.3, as the strain rate increases, when the relative density rises from 0.6 to 0.9, the differences in the tangent modulus during the first stage of the stress–strain curve, the stress differences at the same strain, and the differences in pre-consolidation pressure evolve from negative values to approximately zero and then to positive values, increasing continuously. Specifically, the differences in the tangent modulus are approximately −214 MPa, −21 MPa, 149 MPa, and 122 MPa, respectively, showing an overall increasing trend. Similarly, the differences in pre-consolidation pressure are approximately −2.2 MPa, 0.99 MPa, 4.9 MPa, and 2.9 MPa, respectively, also indicating a continuous increase. It should be noted that for the pre-consolidation point ‘Y’, although in many cases, point ‘Y’ may appear before the stress equilibrium is reached in the specimen, when point ‘Y’ emerges, the stress difference between the front and rear ends of the specimen is not significant, and this difference has a substantially similar impact on the yield point ‘Y’ under all testing conditions. This variation in pre-consolidation pressure is consistent with the results reported in the related literature [22]. Additionally, the stress differences at the same strain during the second stage shift from negative to positive, also increasing continuously. The differences between peak strains are approximately 0.6%, −0.72%, −2.16%, and −2.6%, respectively, showing a decreasing trend. These observations suggest that the bearing capacity of the specimens transitions from decreasing to increasing, with the degree of improvement continually rising. The primary reason for this phenomenon is that, at a moisture content of 0.3, specimens with a relative density of 0.9 exhibit higher saturation compared to those with a relative density of 0.6, approaching complete saturation. This results in lower friction resistance between particles, with less impact energy consumed by deformations caused by skeletal slip and rotation, leaving more energy for particle breakage. Furthermore, at lower strain rates, specimens with higher saturation exhibit more complete stress transmission and better deformation integrity, allowing more low-strength particles to participate in breakage, thereby reducing the bearing capacity of the specimens. However, specimens with higher relative density have smaller porosity ratios, larger coordination numbers of particles, and larger contact areas between particles, along with higher saturation levels. This leads to more uniform stress transmission and smaller contact stresses under the same conditions, making it less likely to reach the critical stress for particle breakage. This effect increasingly contributes to specimen deformation as the strain rate increases. Meanwhile, with the elevation of strain rate, the inertia effect becomes increasingly prominent, leading to a heightened localization of deformation. Consequently, the contribution of water lubrication diminishes progressively, while the degree of water medium participation in compression augments. However, the compression modulus of water is much larger than that of the coral skeleton. Therefore, the bearing capacity of the specimens transitions from decreasing to increasing, with the degree of improvement continually rising.

Based on the analysis of Figure 6, Figure 7, Figure 8 and Figure 9 and Table 2, it is evident that under different conditions of relative density, despite significant variations in initial porosity ratios, the strains corresponding to the yield points generally remain within 2%. During the initial stage of compression, the compression curves exhibit a relatively gentle slope, with minimal changes in porosity ratio, indicating that only a small fraction of particles undergo positional rearrangement under applied pressure. Upon reaching the pre-consolidation pressure that triggers particle breakage, the porosity ratio decreases at an increasingly rapid rate, or even abruptly, due to the smaller particles filling the voids surrounding larger particles after breakage, leading to a more densely packed state of the specimen. As the relative density increases, the changes in the pre-consolidation point strain and swelling index do not exhibit a definite regularity. The primary reason for this phenomenon is that, in addition to the relative density, the strain at the pre-consolidation point and swelling index is also influenced by a range of factors characterized by random variability, encompassing particle morphology, particle dimension, and sample preparation processes. When the relative density increases from 0.3 to 0.6, the compression index decreases significantly. As it further increases from 0.6 to 0.9, the compression index either decreases markedly or remains roughly unchanged. These observations suggest that after particle breakage, the relative density has a certain influence on the compression curve of granular materials after pre-consolidation. Generally, the higher the relative density, the flatter the compression curve and the lower the compressibility of the specimen.

Under the conditions of a relative density of either 0.6 or 0.9 and a moisture content of 0.3, no lock-up phenomena occur at a tank pressure of 0.2, 0.4, or 0.6 MPa. However, the lock-up phenomenon is observed at 0.8 MPa for both relative densities. Notably, when the relative density is 0.9, the specimen is only in the transitional stage of the lock-up phenomenon at its maximum strain point. Meanwhile, under the conditions of a relative density of 0.3 and tank pressures of 0.4, 0.6, and 0.8 MPa, the lock-up phenomenon occurred at a moisture content of 0.5. However, no lock-up phenomenon was observed at moisture contents of 0 (i.e., dry), 0.1, and 0.2. These findings indicate that higher moisture content, lower relative density, and a larger strain rate tend to increase the probability of the occurrence of the lock-up phenomenon. Zhao et al. pointed out that the occurrence of the lock-up phenomenon is related to the plastic compression of the coral sand skeleton, as well as the elastic compression of pore water and solid particles [34]. Under high moisture content conditions, specimens with higher relative density exhibit smaller porosity ratios and higher saturation levels, leading to increased inter-particle friction resistance against displacement among coral sand grains and making particle breakage more difficult. Consequently, the final deformation of the specimen is smaller. Additionally, a lower strain rate, corresponding to lower impact strength, results in less input compression energy, further reducing the final deformation of the specimen. Consequently, the locality of deformation, the degree of plastic compression in the coral sand skeleton, and the extent of elastic compression in solid particles and water media with high compression moduli are also lower. As a result, the lock-up phenomenon is less likely to occur.

## 4. Conclusions

This paper presents dynamic compression tests conducted on coral sand with varying relative densities under quasi-one-dimensional strain conditions and at different strain rates. Preliminary insights into the dynamic response characteristics of coral sand under quasi-one-dimensional shock compression are obtained, and the effect of relative density on these characteristics is analyzed. The main conclusions are as follows:

(1) The stress–strain curves of coral sand exhibit approximately three basic forms. The first form displays a three-stage characteristic: the first stage is characterized by skeletal slippage and yield, with the stress–strain curve and corresponding compression curve being approximately linear; the second stage is the compaction stage, also with an approximately linear stress–strain curve but with a different slope compared to the first stage; and the third stage is the unloading stage, where stress is rapidly released. The second form is similar to the first, also approximately displaying a three-stage characteristic, but differs in that the stress–strain relationship during the second stage approximates an exponential relationship. The third form is also similar to the first but differs in that it approximately exhibits a four-stage characteristic, with an additional lock-up phenomenon stage where the stress–strain relationship first approximates an exponential relationship and then becomes linear. The occurrence of lock-up phenomena is influenced by the combined effects of relative density, strain rate, and moisture content. Higher moisture content, lower relative density, and a larger strain rate tend to facilitate the occurrence of lock-up phenomena.

(2) The stress–strain relationship of the second basic form mainly occurs under conditions of low relative density and no water, while the third basic form mainly appears under conditions of high strain rate and high moisture content. Under other conditions, the stress–strain relationship predominantly follows the first basic form.

(3) The influence of relative density on the compression behavior of coral sand is comprehensively affected by both moisture content and strain rate. Under anhydrous or low saturation conditions, as the relative density increases, both the tangent modulus in the first stage of the stress–strain curve and the pre-consolidation pressure exhibit varying degrees of enhancement, and the bearing capacity of the specimen continues to improve. However, under conditions of high saturation and a low strain rate, an increase in relative density paradoxically results in a decrease in the bearing capacity of the specimen. Nevertheless, as the strain rate continues to increase, the bearing capacity of the specimen gradually improves, swiftly transitioning from a state of decrease to increase, with the magnitude of this improvement continually intensifying.

(4) Under various conditions of relative density, the strains corresponding to the yield points are generally within 2%. During the first stage of impact compression, the void ratio undergoes insignificant changes, and the deformation of the specimens is primarily contributed by the crushing of coral particles. When the relative density increases from 0.3 to 0.6, the compression index decreases significantly. As the relative density further increases from 0.6 to 0.9, the compression index either decreases markedly or remains roughly constant. However, neither the strain corresponding to the yield point nor the swelling index exhibits a clear pattern or regularity.

## Figures and Tables

**Table 1:** Statistics of test conditions.

Test Number (*i* = 1, 2)	Relative Density$/D_{r}$	Moisture Content/w	Gas Storage Tank Pressure/MPa	Test Number	Relative Density$/D_{r}$	Moisture Content/w	Gas Storage Tank Pressure/MPa
0.3-0-0.2-*i*	0.3	0	0.2	0.6-0.3-0.2	0.6	0.3	0.2
0.3-0-0.4-*i*	0.3	0	0.4	0.6-0.3-0.4	0.6	0.3	0.4
0.3-0-0.6-*i*	0.3	0	0.6	0.6-0.3-0.6	0.6	0.3	0.6
0.3-0-0.8-*i*	0.3	0	0.8	0.6-0.3-0.8	0.6	0.3	0.8
0.3-0.5-0.2-*i*	0.3	0.5	0.2	0.6-0.1-0.2	0.6	0.1	0.2
0.3-0.5-0.4-*i*	0.3	0.5	0.4	0.6-0.1-0.4	0.6	0.1	0.4
0.3-0.5-0.6-*i*	0.3	0.5	0.6	0.6-0.1-0.6	0.6	0.1	0.6
0.3-0.5-0.8-*i*	0.3	0.5	0.8	0.6-0.1-0.8	0.6	0.1	0.8
0.3-0.2-0.2-*i*	0.3	0.2	0.2	0.9-0-0.2	0.9	0	0.2
0.3-0.2-0.4-*i*	0.3	0.2	0.4	0.9-0-0.4	0.9	0	0.4
0.3-0.2-0.6-*i*	0.3	0.2	0.6	0.9-0-0.6	0.9	0	0.6
0.3-0.2-0.8-*i*	0.3	0.2	0.8	0.9-0-0.8	0.9	0	0.8
0.3-0.1-0.2-*i*	0.3	0.1	0.2	0.9-0.3-0.2	0.9	0.3	0.2
0.3-0.1-0.4-*i*	0.3	0.1	0.4	0.9-0.3-0.4	0.9	0.3	0.4
0.3-0.1-0.6-*i*	0.3	0.1	0.6	0.9-0.3-0.6	0.9	0.3	0.6
0.3-0.1-0.8-*i*	0.3	0.1	0.8	0.9-0.3-0.8	0.9	0.3	0.8
0.6-0-0.2-*i*	0.6	0	0.2	0.9-0.1-0.2	0.9	0.1	0.2
0.6-0-0.4-*i*	0.6	0	0.4	0.9-0.1-0.4	0.9	0.1	0.4
0.6-0-0.6-*i*	0.6	0	0.6	0.9-0.1-0.6	0.9	0.1	0.6
0.6-0-0.8-*i*	0.6	0	0.8	0.9-0.1-0.8	0.9	0.1	0.8

**Table 2:** Statistics of parameters of compressibility under various test conditions.

Test Number	$C_{c}$	$C_{s}$	*P*_c_/MPa	Test Number	$C_{c}$	$C_{s}$	*P*_c_/MPa	Test Number	$C_{c}$	$C_{s}$	*P*_c_/MPa
0.3-0-0.2-1	0.153/0.225	0.002	/	0.3-0-0.2-2	0.203	0.003	1.49	0.3-0.1-0.8-1	0.211/0.489	0.02	2.14
0.6-0-0.2-1	0.073/0.209	0.008	/	0.6-0-0.2-2	0.082/0.161	0.009	/	0.6-0.1-0.8-1	0.406	0.019	5.51
0.9-0-0.2-1	0.174	0.01	3.75	0.9-0-0.2-2	0.174	0.003	4	0.9-0.1-0.8-1	0.429	0.012	11
0.3-0-0.4-1	0.162/0.287	0.016	1.42	0.3-0-0.4-2	0.184/0.318	0.015	1.78	0.3-0.1-0.8-2	0.112/0.469	0.023	1.65
0.6-0-0.4-1	0.147/0.235	0.012	2.14	0.6-0-0.4-2	0.121/0.2	0.012	1.56	0.6-0.1-0.8-2	0.235/0.568	0.023	4.76
0.9-0-0.4-1	0.123/0.233	0.015	2.9	0.9-0-0.4-2	0.123/0.233	0.008	2.81	0.9-0.1-0.8-2	0.429	0.024	15.54
0.3-0-0.6-1	0.223/0.335	0.02	1.65	0.3-0-0.6-2	0.131/0.278	0.015	/	0.6-0.3-0.2-1	0.22/1.08	0.01	7.61
0.6-0-0.6-1	0.165/0.314	0.011	2.15	0.6-0-0.6-2	0.131/0.257	0.011	2	0.9-0.3-0.2-1	0.077/0.24	0.009	4.79
0.9-0-0.6-1	0.18/0.262	0.016	4.64	0.9-0-0.6-2	0.18/0.262	0.014	4.5	0.6-0.3-0.4-1	0.391	0.012	9.64
0.3-0-0.8-1	0.263/0.309	0.019	1.96	0.3-0-0.8-2	0.154/0.379	0.024	1.46	0.9-0.3-0.4-1	0.269	0.011	9.51
0.6-0-0.8-1	0.176/0.295	0.018	1.79	0.6-0-0.8-2	0.344	0.016	3.92	0.6-0.3-0.6-1	0.501	0.018	11.27
0.9-0-0.8-1	0.198/0.321	0.017	5.26	0.9-0-0.8-2	0.167/0.321	0.013	5.26	0.9-0.3-0.6-1	0.365	0.013	16.08
0.3-0.1-0.2-1	0.173	0.005	1.44	0.3-0.1-0.2-2	0.146	0.004	1.43	0.6-0.3-0.8-1	0.276	0.018	4.95
0.6-0.1-0.2-1	0.196	0.009	3.61	0.6-0.1-0.2-2	0.171	0.003	3.22	0.9-0.3-0.8-1	0.292	0.022	11.68
0.9-0.1-0.2-1	0.112/0.556	0.009	7.15	0.9-0.1-0.2-2	0.151/0.837	0.002	9.4	0.6-0.3-0.2-2	0.16/1.08	0.012	6.4
0.3-0.1-0.4-1	0.221/0.479	0.015	4.11	0.3-0.1-0.4-2	0.356	0.009	4.27	0.9-0.3-0.2-2	0.077/0.24	0.004	4.8
0.6-0.1-0.4-1	0.338	0.015	5.86	0.6-0.1-0.4-2	0.348	0.016	6.67	0.6-0.3-0.4-2	0.326	0.014	8.51
0.9-0.1-0.4-1	0.341	0.01	14.16	0.9-0.1-0.4-2	0.173/0.604	0.014	10.47	0.9-0.3-0.4-2	0.276	0.011	10.63
0.3-0.1-0.6-1	0.164/0.512	0.02	2.16	0.3-0.1-0.6-2	0.165/0.569	0.024	2.91	0.6-0.3-0.6-2	0.533	0.001	12
0.6-0.1-0.6-1	0.478	0.02	8.4	0.6-0.1-0.6-2	0.251/0.519	0.024	6.03	0.9-0.3-0.6-2	0.341	0.009	17
0.9-0.1-0.6-1	0.466	0.019	17.62	0.9-0.1-0.6-2	0.448	0.012	16.61	0.6-0.3-0.8-2	0.395	0.018	9.6
								0.9-0.3-0.8-2	0.253	0.011	8.74

## Data Availability

The original contributions presented in the study are included in the article, further inquiries can be directed to the corresponding author.

## References

[1] Zhang J.M. (2004). Study on the Fundamental Mechanical Characteristics of Calcareous Sand and the Influence of Particle Breakage. Master’s Thesis.

[2] Wang X.Z., Jiao Y.Y., Wang R., Hu M.J., Meng Q.S., Tan F.Y. (2011). Engineering characteristics of the calcareous sand in Nansha Islands, South China Sea. Eng. Geol..

[3] Wang X.Z., Wang X., Jin Z.C., Zhu C.Q., Wang R., Meng Q. (2017). Investigation of engineering characteristics of calcareous soils from fringing reef. Ocean Eng..

[4] Wang X., Wu Y.X., Lu Y., Cui J., Wang X., Zhu C. (2021). Strength and dilatancy of coral sand in the South China Sea. Bull. Eng. Geol. Environ..

[5] Wang X., Wu Y., Cui J., Zhu C.Q., Wang X.Z. (2020). Shape Characteristics of Coral Sand from the South China Sea. J. Mar. Sci. Eng..

[6] Ma L.J., Li Z., Luo Z.M., Wei H.Z., Duan L.Q. (2019). Experimental study of strain rate effects on mechanical properties of coral particles. Chin. J. Rock Soil Mech..

[7] Xiao Y., Yuan Z.X., Chu J., Liu H.L., Huang J.Y., Luo S.N., Wang S., Lin J. (2019). Particle breakage and energy dissipation of carbonate sands under quasi-static and dynamic compression. Acta Geotech..

[8] Xiao Y., Liu H., Xiao P., Xiang J. (2016). Fractal crushing of carbonate sands under impact loading. Géotechnique Lett..

[9] Zhou B., Ku Q., Li C.H., Wang H.B., Dong Y.K., Cheng Z. (2022). Single-particle crushing behaviour of carbonate sands studied by X-ray microtomography and a combined finite-discrete element method. Acta Geotech..

[10] Lv Y.R., Wang C., Huang H.X., Zuo D.J. (2021). Study on particle structure and crushing behaviors of coral sand. Chin. J. Rock Soil Mech..

[11] Ma L.J., Li Z., Wang M.Y., Wei H.Z., Fan P.X. (2019). Effects of size and loading rate on the mechanical properties of single coral particles. Powder Technol..

[12] Wang C.Y., Ding X.M., Xiao Y., Peng Y., Liu H.L. (2021). Effects of relative densities on particle breaking behaviour of non-uniform grading coral sand. Powder Technol..

[13] Chen X., Shen J.H., Wang X., Yao T., Xu D.X. (2022). Effect of Saturation on Shear Behavior and Particle Breakage of Coral Sand. J. Mar. Sci. Eng..

[14] Gao R., Ye J.H. (2023). Mechanical behaviors of coral sand and relationship between particle breakage and plastic work. Eng. Geol..

[15] Wu Y., Cui J., Li N., Wang X., Wu Y.H., Guo S.Y. (2020). Experimental study on the mechanical behavior and particle breakage characteristics of hydraulic filled coral sand on a coral reef island in the South China Sea. Rock Soil Mech..

[16] Bo H. (2008). Research on the Particle Breakage Mechanical Characteristics and Constitutive Model of Calcareous Sand Under Triaxial Conditions. Master’s Thesis.

[17] Yu F.W. (2018). Particle breakage in triaxial shear of a coral sand. Soils Found..

[18] Yu H.Z. (2006). Experimental Research on Dynamic Behavior of Saturated Calcareous Sand under Complex Stress Conditions. Master’s Thesis.

[19] Li J.G. (2004). Experimental Research on Dynamical Behavior of Saturated Calcareous Sand Under Wave Loading. Master’s Thesis.

[20] Wang G., Wang Z.N., Ye Q.G., Zhang J.J. (2021). Particle breakage evolution of coral sand using triaxial compression tests. J. Rock Mech. Geotech. Eng..

[21] Omidvar M., Iskander M., Bless S. (2012). Stress-strain behavior of sand at high strain rates. Int. J. Impact Eng..

[22] Dong K., Ren H.Q., Ruan W.J., Ning H.J., Guo R.Q., Huang K. (2020). Study on strain rate effect of coral sand. Chin. J. Explos. Shock Waves.

[23] Wei J.Q., Wang M.Y., Qiu Y.Y., Zhao Z.Y. (2018). Impact compressive response of calcareous sand. Chin. J. Vib. Shock.

[24] Wei J.Q., Lv Y.R., Liu G.Q., Zhang L., Li L. (2019). One-dimensional impact responses and energy absorption of calcareous sand. Chin. J. Rock Soil Mech..

[25] Wen Z., Qiu Y.Y., Zi M., Zhao Z.Y., Wang M.Y. (2019). Experimental study on quasi-one-dimensional strain compression of calcareous sand. Chin. J. Explos. Shock Waves.

[26] Lv Y.R., Wang M.Y., Wei J.Q., Liao B. (2018). Experimental techniques of SHPB for calcareous sand and its dynamic behaviors. Chin. J. Explos. Shock Waves.

[27] Lv Y.R., Li Z.Z., Li L. (2019). One-dimensional compression behavior of calcareous sand and its experimental technology under high stress conditions. Chin. J. Rock Mech. Eng..

[28] Lv Y.R., Liu J.G., Xiong Z.M. (2019). One-dimensional dynamic compressive behavior of dry calcareous sand at high strain rates. J. Rock Mech. Geotech. Eng..

[29] Li X., Lv Y.R., Su Y.C., Zou K.H., Wang Y., Huang W.X. (2023). Coupling effects of morphology and inner pore distribution on the mechanical response of calcareous sand particles. J. Rock Mech. Geotech. Eng..

[30] Lv Y.R., Li X., Fan C.F., Su Y.C. (2021). Effects of internal pores on the mechanical properties of marine calcareous sand particles. Acta Geotech..

[31] Lv Y.R., Wang Y., Zuo D. (2019). Effects of particle size on dynamic constitutive relation and energy absorption of calcareous sand. Powder Technol..

[32] Lv Y.R., Li X., Wang Y. (2020). Particle breakage of calcareous sand at high strain rates. Powder Technol..

[33] Lv Y.R., Hu J.M., Zhang D.D., Wang Y., Su Y.C. (2024). Particle breakage of calcareous sand from low-high strain rates. J. Rock Mech. Geotech. Eng..

[34] Zhao Z.Y., Qiu Y.Y., Zi M., Xing H.D., Wang M.Y. (2020). Experimental study on dynamic compression of unsaturated calcareous sand. Chin. J. Explos. Shock Waves.

[35] Song B., Chen W.N., Luk V. (2009). Impact compressive response of dry sand. Mech. Mater..

[36] Huang J., Xu S., Hu S. (2013). Effects of grain size and gradation on the dynamic responses of quartz sands. Int. J. Impact Eng..

[37] Luo H.Y., Cooper W.L., Lu H.B. (2014). Effects of particle size and moisture on the compressive behavior of dense Eglin sand under confinement at high strain rates. Int. J. Impact Eng..

[38] Wang W., Zhang Z.H., Huo Q., Song X.D., Yang J.C., Wang X.F., Wang J.H., Wang X. (2022). Dynamic Compressive Mechanical Properties of UR50 Ultra-Early-Strength Cement-Based Concrete Material under High Strain Rate on SHPB Test. Materials.

[39] Zhang H., Ren H.Q., Mu C.M., Wu X.Y., Huang K., Wang F. (2023). Experiment Study on the Influence of Density and Confining Pressure on Triaxial Shear Properties of Calcareous Sand. Materials.

[40] Ravi-Chandar K., Ma Z. (2000). Inelastic Deformation in Polymers under Multiaxial Compression. Mech. Time-Depend. Mater..

[41] Veyera G.E. (1994). Uniaxial Stress-Strain Behavior of Unsaturated Soils at High Strain Rates: WR-TL-93-3523.

[42] Barr A.D., Clarke S.D., Tyas A., Warren J.A. (2018). Effect of moisture content on high strain rate compressibility and particle breakage in loose sand. Exp. Mech..

